# Bilateral blindness following Russell’s viper bite - a rare clinical presentation: a case report

**DOI:** 10.1186/1752-1947-8-99

**Published:** 2014-03-24

**Authors:** Chandrika Jayakanthi Subasinghe, Chamara Sarathchandra, Thambyaiah Kandeepan, Aruna Kulatunga

**Affiliations:** 1 General Medical Unit, National Hospital of Sri Lanka, Colombo, Sri Lanka

**Keywords:** Blindness, Russell’s viper, Ischemic stroke

## Abstract

**Introduction:**

Russell’s viper (*Daboia russelii)* is one of the most common medically important snakes reported in Sri Lanka. Its envenomation leads to significant mortality and morbidity with local, hematological, neurological and renal complications. Here we report the case of a patient who presented with bilateral blindness secondary to a bilateral posterior circulation ischemic stroke instead of the usual neurological manifestations of Russell’s viper envenomation. There were no reported cases of cortical blindness following a Russell’s viper bite. Only a few reported cases of ischemic strokes following a Russell’s viper bite were found in the literature.

**Case presentation:**

A 54-year-old Sri Lankan woman developed bilateral blindness due to a posterior circulation infarct following Russell’s viper envenomation.

**Conclusion:**

Ischemic stroke following a Russell’s viper bite is very rare and cortical blindness is not reported as the clinical presentation. Also, we emphasize the importance of considering the possibility of ischemic stroke in patients who develop unusual neurological manifestations following Russell’s viper envenomation.

## Introduction

Russell’s viper (*Daboia russelii)* envenomation is common in Sri Lanka [1], and is characterized by coagulopathy (77%), local swelling (92%), renal involvement (18%) and neurotoxicity (78%) [2]. Russell’s viper is the offending agent in the majority of snake bite cases (48%) with neurotoxic envenomation in Sri Lanka [3]. Among neurological manifestations, ptosis was the most common manifestation seen in 85.7% of patients, followed by ophthalmoplegia (75%), limb weakness (26.8%), respiratory failure (17.9%), palatal weakness (10.7%), neck muscle weakness (7.1%) and delayed sensory neuropathy (1.8%) [3]. Blindness due to spitting of venom into the eyes by spitting snakes and rare cases of vascular retinal changes and optic neuritis have been reported in Africa and India [4-6]. There were no reported cases of cortical blindness following a Russell’s viper bite. Only a very few reported cases of ischemic strokes following a Russell’s viper bite from Sri Lanka and worldwide were found in the literature [7-9] (Table 1).

**Table 1:** Previously published cases of ischemic strokes following Russell’s viper bites

**Cases of ischemic strokes**	**Stroke**	**Clinical presentation**
Ameratunga B: **Middle cerebral occlusion following Russel’s viper bite.***J Trop Med Hyg* 1972, **75**:95–97 [8].	Middle cerebral artery occlusion	Not known
Gawarmmana I, Mendis S, Jeganathan K: **Acute ischaemic stroke due to bites by** ***Daboia russelii*** **in Sri Lanka – first authenticated case series.***Toxicon* 2009, **54**:421–428 [7].	Cerebellum, Bilateral frontal and parietal lobes	Low GCS
	R deep parietal and lentiform nucleus	Left hemiparesis
	R frontal and R cerebellum	Left hemiparesis
	Left caudate and bilateral occipital lobes	Low GCS, Convulsions
	L and R middle cerebral artery territory and bilateral occipital lobes	Low GCS, Convulsions
	Multiple bilateral cortical and cerebellum	Low GCS, Motor weakness
	Left frontal lobe	Expressive dysphasia
	multiple - cerebellum and occipital lobes	Low GCS
	R parietal and temporal lobes	Left hemiparesis
Narang SK, Paleti S, Azeez Asad MA, Samina T: **Acute ischemic infarct in the middle cerebral artery territory following a Russell's viper bite.***Neurol India* 2009, **57**: 479–480 [9].	L middle cerebral infarct	Expressive dysphasia

GCS, Glasgow Coma Scale; L, Left; R, Right.

## Case presentation

A 54-year-old healthy Sri Lankan woman presented 30 minutes following a Russell’s viper bite. The snake was brought in and identified. On her admission to our facility, a 20-minute whole blood clotting test (20-WBCT) was prolonged with clinical hematuria without other obvious bleeding manifestations. Local envenomation was apparent with edema at the bite site but there were no features of neurotoxicity. Initially, 10 vials of anti-snake venom serum were given and the hematuria eased. In three to four hours, our patient complained of visual blurring followed by bilateral visual loss. There was no ptosis or ophthalmoplegia. Fundoscopic examination and slit lamp examination of her retinae were completely normal but she could perceive only light. There were no other neurological manifestations.

**Figure 1:**
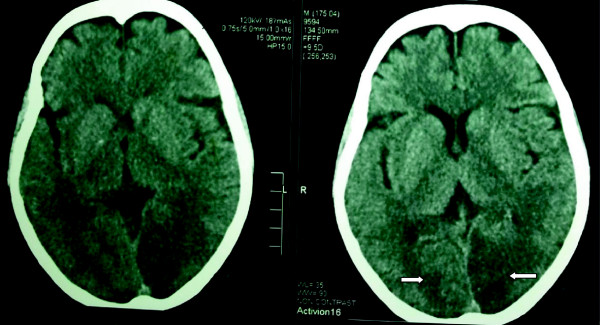
Non-contrast computed tomography of the brain showing bilateral occipital infarcts.

A non-contrast computed tomograph (CT) of her brain showed bilateral posterior circulation infarcts without hemorrhages (Figure 1). The 20-WBCT, prothrombin time, thrombin time and partial thromboplastin time with kaolin tests were initially prolonged but normalized the following day. Her D-dimer level was slightly elevated. Electrocardiogram (ECG), transthoracic echocardiogram, lipid profile, fasting plasma glucose and duplex scan of vertebral arteries were all normal. Her serum creatinine was monitored periodically and remained normal throughout.

She received anti-venom serum initially and supportive care. Management options for occipital infarcts were minimal since acute thrombolysis was contraindicated in this setting. She underwent the usual stroke work-up, education, counseling and follow-up for rehabilitation. At the three-month follow-up she showed mild improvement of visual acuity and she was able to count fingers at 1 meter distance.

## Discussion

A Russell’s viper bite is a common cause of morbidity and mortality in Sri Lanka. Neurotoxity is well known but the usual neurological manifestations are due to transient presynaptic inhibition of neuromuscular transmission of secretory phospholipase A2, which improves with antivenom serum [10]. Blindness due to rare cases of vascular retinal changes and optic neuritis following viper bites has been reported in Africa and India [4-6]. Our patient developed cortical blindness due to bilateral, multiple, large artery territory ischemic infarcts with normal funduscopic findings. We postulated a generalized procoagulant effect of snake venom causing thrombosis of multiple large vessels as the underlying pathophysiology. She had other evidence for generalized coagulopathy, procoagulant effect and fibrinolysis, such as a prolonged clotting profile and elevated D-dimer levels. Multiple infarcts favor generalized pathology rather than local atherosclerotic thrombosis. Other sources of thromboembolism were excluded. As our patient developed a stroke within hours of the bite, vasculitis does not fit into the picture. She did not have hypotension following the bite or during anti-venom treatment and territories of infarct did not lie in watershed areas of vascular supply.

Basic pro-coagulation metalloprotease (Russell's viper basic coagulant metalloprotease) [11], procoagulant factors V and X, protease and phospholipase A2 [12] in Russell’s viper venom have been demonstrated to have procoagulant effects. Russell’s viper bite-associated thrombosis in multiple organs, such as the heart and limbs has been well reported in the literature [13]. Russell’s viper venom has also been used to trigger arterial thrombosis in a model using atherosclerotic rabbits to study the mechanism of arterial thrombosis [14,15].

## Conclusion

Ischemic stroke is rare following snake bite, although theoretical possibilities of infarction as well as hemorrhage are well explained. The dose of venom, species difference in different areas and the patient’s co-morbidities may decide the occurrence of infarcts. Also silent, small infarcts may go clinically unnoticed. Therefore, we emphasize the importance of considering the possibility of ischemic stroke in patients who develop unusual neurological manifestations following snake bite. In addition to the acute management with antivenom serum and supportive care, these patients need long-term follow-up and rehabilitation.

## Consent

Written informed consent was obtained from the patient for publication of this case report and accompanying images. A copy of the written consent is available for review by the Editor-in-Chief of this journal.

## Abbreviations

20-WBCT: 20-minute whole blood clotting test; CT: computed tomography; ECG: electrocardiogram.

## Competing interests

The authors declare that they have no competing interests.

## Authors’ contributions

Analysis and interpretation of patient data and the literature review were done by CJS, CS and TK. AK guided the other authors in reporting this case and corrected the final manuscript. All authors were involved in the management of the patient and read and approved the final manuscript.

## Authors’ information

CJS (MBBS) is a registrar in medicine. CS (MBBS, MD) is a senior registrar in medicine. TK (MBBS, MD) is a senior registrar in medicine. AK (MBBS, MD, FRCP) is a senior consultant physician.
